# Investigating the role of visual and auditory search in reading and developmental dyslexia

**DOI:** 10.3389/fnhum.2013.00597

**Published:** 2013-09-25

**Authors:** Marie Lallier, Sophie Donnadieu, Sylviane Valdois

**Affiliations:** ^1^Basque Center on Cognition Brain and LanguageDonostia-San Sebastian, Spain; ^2^Laboratoire de Psychologie et NeuroCognition, UMR CNRS 5105Grenoble-Chambéry, France; ^3^Faculté de Psychologie, Université de SavoieChambéry, France; ^4^Centre National pour la Recherche ScientifiqueParis, France

**Keywords:** dyslexia, reading, visual search, auditory search, attention, temporal processing, visual attention span, phonology

## Abstract

It has been suggested that auditory and visual sequential processing deficits contribute to phonological disorders in developmental dyslexia. As an alternative explanation to a phonological deficit as the proximal cause for reading disorders, the visual attention span hypothesis (VA Span) suggests that difficulties in processing visual elements simultaneously lead to dyslexia, regardless of the presence of a phonological disorder. In this study, we assessed whether deficits in processing simultaneously displayed visual or auditory elements is linked to dyslexia associated with a VA Span impairment. Sixteen children with developmental dyslexia and 16 age-matched skilled readers were assessed on visual and auditory search tasks. Participants were asked to detect a target presented simultaneously with 3, 9, or 15 distracters. In the visual modality, target detection was slower in the dyslexic children than in the control group on a “serial” search condition only: the intercepts (but not the slopes) of the search functions were higher in the dyslexic group than in the control group. In the auditory modality, although no group difference was observed, search performance was influenced by the number of distracters in the control group only. Within the dyslexic group, not only poor visual search (high reaction times and intercepts) but also low auditory search performance (d′) strongly correlated with poor irregular word reading accuracy. Moreover, both visual and auditory search performance was associated with the VA Span abilities of dyslexic participants but not with their phonological skills. The present data suggests that some visual mechanisms engaged in “serial” search contribute to reading and orthographic knowledge via VA Span skills regardless of phonological skills. The present results further open the question of the role of auditory simultaneous processing in reading as well as its link with VA Span skills.

## Introduction

Developmental dyslexia is a neurocognitive disorder reflected by severe and persistent reading difficulties in individuals who have been provided with appropriate schooling, present a non-verbal IQ within the normal range, and do not suffer from any sensory or psychiatric disorders. A number of neuroimaging and behavioral studies now suggest that reading difficulties in dyslexia may not stem from a unique but rather multiple origins (Ramus and Ahissar, 2012; Koyama et al., 2013; van Ermingen-Marbach et al., 2013). Developmental dyslexia in this context is seen as a multifactorial and heterogeneous disorder. For example, the visual attention span (VA Span, hereafter) hypothesis describes at least two cognitive impairments (phonological and visual attentional) that can equally but independently lead to developmental dyslexia (Bosse et al., 2007). Looking at two large samples of French and English dyslexic children, Bosse et al. (2007) report that the reading difficulties of dyslexic children were either accompanied by a single phonological disorder (i.e., phonological awareness, phonological short term memory, phonological fluency), a single VA Span deficit (without phonological problems), or a combination of those two. Importantly, Peyrin et al. (2012) found that the biological bases for those two dyslexic cognitive subtypes were independent: they found that a dysfunction located within the left inferior frontal gyrus characterized dyslexia associated with a phonological disorder whereas a dysfunction of the superior parietal lobules bilaterally was seen in the VA Span dyslexic subtype.

The VA Span is defined as the number of visual elements that can be processed simultaneously (at a glance) in a visual multi-element array, regardless of the verbal or non-verbal nature of those elements (Lobier et al., 2012). VA Span skills are thought to tap into perceptual attention (i.e., attention skills which enhance perceptual encoding and its clarity) and be specifically critical for (i) processing simultaneously all the letters within whole-word visual forms, (ii) building-up lexical orthographic knowledge and (iii) enhancing the recognition of previously unfamiliar words (Bosse and Valdois, 2009; Bosse et al., 2013). Moreover, VA Span skills have been shown to play a significant role at various stages of typical reading development by contributing to reading variance independently from phonological skills (Bosse and Valdois, 2009).

For the VA Span hypothesis, the simultaneous dimension of visual perceptual attention plays a central role in reading development independently of phonology. Contrastively, the sequential dimension of visual perceptual attention has been proposed as a significant contributor to dyslexia associated with phonological difficulties (sluggish attentional shifting theory of dyslexia, Hari and Renvall, 2001). Supporting the claims of both the VA Span and the sluggish attentional shifting theories, Lallier et al. (2010a) showed that a dyslexic adult with a severe phonological deficit but preserved VA Span skills was impaired on visual sequential attentional skills. This suggests that phonological, and visual sequential processing disorders can co-occur in dyslexia regardless of visual simultaneous processing problems, i.e., VA Span deficits. Along the same lines, some studies showed that dyslexic participants exhibited visual impairments on paradigms where stimuli were presented sequentially but not simultaneously (Ben-Yehudah and Ahissar, 2004; Conlon et al., 2004; Ram-Tsur et al., 2006), or the opposite (Yap and Van der Leij, 1993; Lassus-Sangosse et al., 2008).

These studies suggest that the dissociation between sequential and simultaneous visual processing deficits in dyslexia essentially depend on the stimulus presentation mode of the task. However, the link between sequential presentation paradigms and the sequential dimension involved in ecologic reading is rather indirect: orthographic units never appear and disappear sequentially at a unique fixation point (*externally driven* sequential processing). Rather, the self-paced visual attentional captures within and between words generate the sequential dimension present in the reading activity (*internally driven* sequential processing). Conversely, simultaneous visual processes such as VA Span skills are directly involved in ecologic reading since they reflect visual attention skills at play during an ocular fixation (Prado et al., 2007).

Visual search paradigms have been proposed to reflect both sequential (Vidyasagar and Pammer, 1999, 2010; Vidyasagar, 2004) and simultaneous (Marendaz et al., 1996) visual perceptual attention at play in reading. In those paradigms, participants are presented with a stimulus display where a target presented simultaneously with a set of distracters has to be detected as fast as possible. Within the framework of the “Feature Integration Theory” (Treisman and Gelade, 1980) two types of search tasks, in which the type of stimuli presented varies, are generally administered and require distinct visual processes. In the so-called “parallel” search, the target possesses only one feature which differentiates it from all the distracters (e.g., “Q” among “O”s). In this condition reaction times (RT) for target detection are not affected by the number of distracters: a pre-attentive “pop-out” effect for the target occurs because a battery of visual analyzers, specialized for detecting that unique feature, automatically captures the attentional focus. In the so-called “serial” search, the target is characterized by the conjunction of two features (e.g., “O” among “Q”s). In that case, RTs for target detection increase linearly as a function of the number of distracters because an effortful sequential screening, thought to engage controlled attention, occurs to search for the target. When assessed on visual search paradigms, dyslexic children (Casco and Prunetti, 1996; Marendaz et al., 1996; Vidyasagar and Pammer, 1999) and adults (Iles et al., 2000; Buchholz and McKone, 2004; de Boer-Schellekens and Vroomen, 2012) are repeatedly found to be impaired on the “serial” search condition, suggesting a visual attention deficit in this population. Typically, dyslexic participants present a higher search slope coefficient than skilled readers, indicating that they process a smaller amount of stimuli per second in the display.

To explain these deficits, two hypotheses regarding the nature of visual attention problems have been suggested: Marendaz et al. (1996) suggest the hypothesis of a reduction of the number of elements that dyslexic individuals can encode simultaneously under fixation whilst searching for the target (i.e., reduced VA Span). Alternatively and according to the feature integration theory of visual search, Vidyasagar (2004, Vidyasagar and Pammer, 1999), proposes that reading problems and difficulties on the visual “serial” search task in dyslexia are both caused by a failure in monitoring sequential spatial attentional shifts under fixation (see also Franceschini et al., 2012). This idea finds support from the neurophysiology of the visual system and the fact that visual information flux arriving from the retina to the visual primary cortex separates into two cortical pathways: (i) the dorsal or “magnocellular” pathway subtending fast/transient processing and object motion encoding and (ii) the dorsal or “parvocellular” pathway subtending slow/sustained visual processing and object identification mechanisms. According to Vidyasagar, the dorsal stream monitors rapid spatial attentional shifts screening serially each of the 7 or 8 letters falling under fixation, therefore facilitating their identification by the ventral system. Like the VA Span hypothesis, this proposal suggests that the key mechanism of visual attention for reading acquisition would occur within an ocular fixation and regardless of the phonological skills of participants (see Pammer et al., 2004, 2005; Vidyasagar and Pammer, 2010)^1^.

In the present study, we present dyslexic and age-matched skilled reader children with a visual task and an auditory one that involve the simultaneous presentation of multiple stimuli. Our first aim was to determine whether deficits classically observed on the “serial” search task in developmental dyslexia were restricted to dyslexia associated with VA Span deficits. Our second aim was to investigate whether any impairment observed on the visual “serial” search task in dyslexia would also occur on an auditory search task. Indeed, since reading requires multimodal resources, it would not be surprising if perceptual attentional deficits in dyslexia were not restricted to only one sensory modality, but also tapped into an amodal pool of resources (Facoetti et al., 2003, 2005, 2010; Lallier et al., 2009, 2010a,c). Moreover, a perceptual asymmetry, which is similar to the one found in visual search tasks, takes place in auditory search tasks. Cusack and Carlyon (2003) presented participants with a task in which a frequency modulated (FM) sound had to be detected among non-modulated sounds (steady sounds), and a task in which the opposite had to be done. No auditory pop-out effect was found for either of the two tasks; however, the participants' accuracy in detecting the FM sound among steady sounds was less affected by the number of distracters than their ability to accurately detect the steady sound among FM distracters was. The authors concluded that auditory search reflected systems specialized for certain auditory features, as well as the limited capacity of attentional resources to process the auditory set, and that the two conditions engaged various degrees of difficulty.

We reasoned that if search mechanisms require simultaneous perceptual attention, and simultaneous perceptual attention reflected in VA Span skills taps into an amodal pool of resources, poor VA Span skills should be associated with poor visual and auditory search performance. Support for this hypothesis comes from a recent study showing that dyslexic children with a VA Span disorder were impaired on simultaneous auditory attention assessed in a dichotic listening task designed to be comparable to the task measuring VA Span abilities (Lallier et al., 2012). Also found that simultaneous auditory attention was unrelated to the phonological awareness and short term memory skills of the participants. Here, we therefore expected that if detecting an auditory target presented simultaneously among auditory distracters involves simultaneous perceptual attention, performance should not be related to the phonological abilities of participants but rather to their VA Span skills.

## Materials and methods

### Participants

Thirty-two French children took part in the present study. A group of 16 dyslexic children (10 boys) was compared to a group of 16 control children (3 boys). All children attended school regularly and had French as native language. They had normal or corrected-to-normal vision, normal hearing level, and no history of neurological or psychiatric disorders.

The 16 dyslexic children were recruited at the “Reference Center for Specific Learning Disorders” of the Pediatric Department of the Hospital of Grenoble and the Neuropediatric Department of the Kremlin-Bicêtre Hospital in Paris where the diagnosis of developmental dyslexia was primarily established by practitioners in charge (i.e., neuropsychologists or neuropediatricians) using both inventories and testing procedures in accordance with the guidelines of the ICD-10 classification of Mental and Behavioral disorders. All the dyslexic participants had normal IQ (full IQ superior to 85 on the WISC-III or WISC-IV, or a score superior to the 25th percentile on the Raven's Progressive Matrices; Raven et al., 1998). Although the two groups were matched for age [controls: 128 ± 5 months; dyslexics: 133 ± 10 months, *t*_(30)_ = 1.7, *p* = 0.09], control children were older regarding reading age [139.2 ± 16 months; dyslexics: 85.7 ± 6.4 months, *z* = 4.8, *p* < 0.001] as measured by the “Alouette” reading test (Lefavrais, 1967).

### Reading skills assessment in the control and the dyslexic children

Reading performance of the 32 participants were assessed using reading lists including a list of 20 words, a list of 20 irregular words and a list of 20 pseudowords, taken from the ODEDYS battery (Jacquier-Roux et al., 2002). Items between lists were matched for letter and syllable lengths, grammatical class and frequency. The 20 pseudowords were legal pseudowords without lexical neighbors. Participants were instructed to read aloud each of the three lists as quickly and as accurately as possible. Both accuracy and reading rate were taken into account.

### Phonological and VA span skills screening of the dyslexic children

Dyslexic children were presented with some additional tasks in order to determine the cognitive disorder associated to their dyslexia at the individual level. Phonological processing was quantified with two tasks: a phonemic deletion task (phonemic awareness) and a pseudoword repetition task (phonological short-term memory). The two phonological tasks were taken from the EVALEC battery (Sprenger-Charolles et al., 2005) in which pseudowords are presented to participants through headphones. The visual whole report and visual partial report tasks (e.g., Bosse et al., 2007; Bosse and Valdois, 2009) were further administered to dyslexic children in order to quantify their VA Span skills.

#### Phonemic awareness

Twelve pseudowords with a tri-phonemic consonant-consonant-vowel structure (CCV) were presented to the children via headphones. The children were instructed to remove (“eat”) the first sound of the pseudoword and say the remaining part. The score corresponded to the percentage of correct answers.

#### Phonological short-term memory

Children were asked to repeat pseudowords as accurately as possible without any time constraint. The task included 24 pseudowords varying in length from three to six syllables. The score corresponded to the percentage of pseudowords accurately repeated.

#### VA span skills

Prior to the visual whole report task, children were administered a control letter identification task. Children were presented with a single letter (each of the 10 consonants presented for the two report tasks described below) in the center of the screen during varying durations (33, 50, 67, 84, and 101 ms) immediately followed by a mask. They were asked to name the letter immediately after being presented.

The whole report task included 20 black consonant strings (composed of 10 consonants, upper-case Arial font, 18 pt). The center-to-center distance between each adjacent consonant was 1.2° so that lateral masking effects were minimized. Stimuli did not include the same letter twice and were not French word skeletons (e.g., C M P T R for “compter”). At the start of each trial, a central fixation point was displayed for 1000 ms followed by a blank screen for 50 ms. Consonant strings were presented horizontally during 200 ms at the center of the screen. Immediately after the string presentation, participants had to recall as many letters as possible. The score corresponded to the percentage of letters accurately reported (identity not location).

In the partial report task, participants were required to orally report a single cued letter presented before briefly within a 5-consonant string. Fifty 5-letter strings were built from the same 10 consonants used in the whole report condition and with the same characteristics as the whole report task. The probe indicating the letter to be reported was a vertical bar presented for 50 ms, 1° below the target letter presented in the string. Each letter was used as target once in each position. Like in the whole report task, a central fixation point was presented for 1000 ms followed by a blank screen for 50 ms. The 5-letter string was then presented at the center of the screen for 200 ms. At the offset of the letter string, the bar probe appeared for 50 ms. Participants were asked to report the cued letter only and to be as accurate as possible and no time pressure. The score corresponded to the percentage of cued letters accurately reported.

### Visual and auditory search tasks

The two search tasks used in the present study were created from two tasks in the visual (Marendaz et al., 1996) and the auditory (Cusack and Carlyon, 2003, Experiment 3) modalities which previously showed a perceptual asymmetry for search performance as a function of target type and number of distracters.

#### Visual stimuli

As in Marendaz et al. (1996), the visual search configurations were composed of two types of capital letters (O and Q, Helvetica font, 28 pts) subtending an average angular size of 0.8°. The letter search display covered a surface of 10.2° by 11.4° (height and width) from a viewing distance of 45 cm. The minimum distance separating two stimuli was never twice as much as the letter size in order to avoid grouping effects. In the “parallel” search condition, children had to detect the target letter “Q” among distracter letters “Os” whereas in the “serial” search condition, they had to detect the target letter “O” among distracter letters “Qs.”

#### Auditory stimuli

Following Experiment 3's procedure of Cusack and Carlyon (2003), the auditory search configurations were composed of 250 ms-long tones randomly distributed over a 1-s window^2^ and across a logarithmic frequency scale (262–4192 Hz), with the constraint that two simultaneously occurring tones had to be separated from at least one third of an octave. In a first condition, children had to detect a FM tone target (described as a “moving sound”), which was modulated at a depth of 5% (0.84 semitones) among pure tones, and in a second condition, they had to detect a steady tone (described as a “non-moving sound”) within FM tones. The sounds were presented over headphones (Earthquake, TS 800) at a level of 70 dB SPL approximately.

#### Procedure

Henceforth, the two search conditions for each modality will be referred to as the “O target” and “Q target” conditions in the visual modality, and “FM target” and “Steady target” conditions in the auditory modality. In both the visual and the auditory tasks, eight search configurations of 4, 10, and 16 stimuli were created, yielding a total of 48 trials for each condition. For each condition, the configurations with various stimulus set sizes were presented randomly and the target was present in half of the trials. Children were instructed to determine whether the target was present or not: in the visual task, they had to press “P” on the keyboard as fast they could as soon as they detected the target, or press “A” when they did not detect any target. In the auditory task, they were instructed to wait until the 1-s auditory configuration finished before pressing the response key when a question about the presence or absence of the target appeared on a white screen.

**Figure 1:**
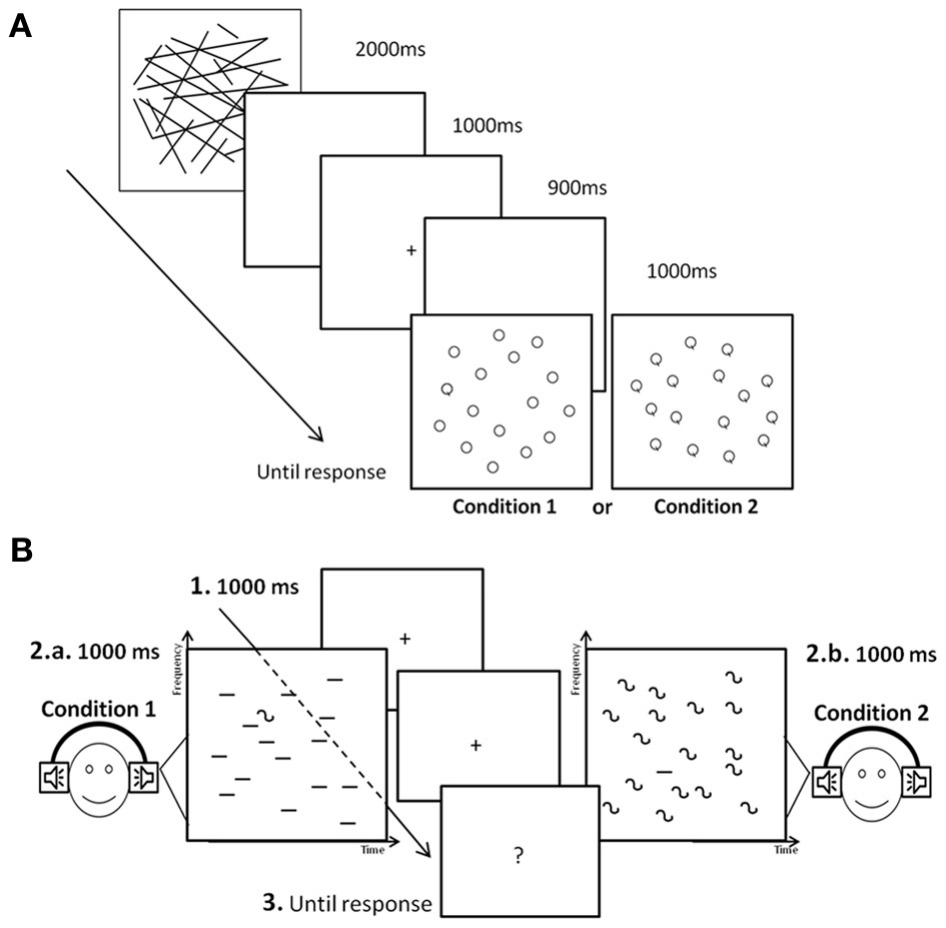
**Illustration of the visual (A) and the auditory (B) search tasks**.

In the visual modality (Figure 1A), children were first presented with a mask subtending the size of the following letter display for 2000 ms. Then, a blank screen was presented for 1000 ms followed by a fixation cross at the center of the screen for 900 ms. A blank screen then appeared for 200 ms and one of the eight visual configurations was presented. After the response of the subject a blank screen then appeared for 1000 ms before the presentation of the mask of the following trial. In the auditory modality (Figure 1B), a fixation cross appeared on a blank screen for 1000 ms and the auditory search configuration was displayed for 1000 ms whilst the cross remained on the screen. After the auditory sequence, a question appeared on the screen asking whether the target was present or not. Before the auditory search task, an identification task composed of 40 trials was administered to the children in order to make sure they could identify both types of sound. On this control task, all children were at ceiling, demonstrating good discrimination skills between FM and steady sounds. In both modalities, a training phase composed of 8 trials was administered prior to the test. The order of administration of the two conditions was counterbalanced between participants, as well as the order between the visual and the auditory tasks.

### Data analysis

Group differences on reading accuracy and speed were assessed by means of independent parametric *t*-tests (or non-parametric *U*-tests when the conditions for carrying out parametric analysis were not assumed) with group (control, dyslexic) as the between-subjects factor. Individual reading performance was compared to age-matched norms from which individual and group average *z*-scores were computed (Bosse and Valdois, 2009). Regarding the cognitive skills of the dyslexic children, individual *z*-scores were computed according to the age-matched corresponding norms for the two phonological tasks (Sprenger-Charolles et al., 2005) and for the VA Span tasks (Bosse and Valdois, 2009).

For the visual and the auditory search task separately, RTs for trials where the target was present and correctly detected, and d' scores were analyzed by means of mixed ANOVAs with group (control, dyslexic) as the between-subjects factor, and condition (O target/Q target; FM target/Steady target) as well as stimulus set size (4, 10, 16) as within subject factors. *Post-hoc* analyses were conducted using Bonferroni tests. In the case of non-homogeneity of variance or non-sphericity of the data, data transformation or Greenhouse-Geisser correction, respectively, were performed.

For partial correlation analyses (controlling for chronological age), we computed an additional search measure corresponding to the average measure of the search performance across the three stimulus set sizes for each modality.

## Results

### Reading skills

As shown in Table 1, the performance of control children was significantly higher than the performance of dyslexic children on the three reading lists (for all *t* or *z* values, *ps* < 0.001, Table 1). All control children performed well within the norm, with all individual *z*-scores being above -1 on all the reading measures. The severe reading difficulties of the dyslexic children were illustrated by an average performance 2 SD below the norm on all the reading measures. Overall, the dyslexic group of the present study exhibited difficulties on both the global (irregular word reading) and analytic (pseudoword reading) reading procedures.

**Table 1:** **Reading skills of the control group (*n* = 16) and the dyslexic group (*n* = 16)**.

	**Control group**		**Dyslexic group**		**Group effect^b^**
	**M (SD)**	***z*-score^a^**	**M (*SD*)**	***z*-score^a^**	
**REGULAR WORDS**					
Accuracy/20	18.8 (1.7)	0.04	13.9 (3.1)	−2.00^*^	*t*_(30)_ = −5.2^***^
Speed (s)	16.8 (4.8)	0.24	60.2 (36.5)	−4.70^***^	*z* = 4.5^***^
**IRREGULAR WORDS**					
Accuracy/20	17.6 (2.1)	0.58	7.1 (3.1)	−2.30^*^	*t*_(30)_ = −10.8^***^
Speed (s)	19.1 (5.9)	0.24	69.3 (37.6)	−4.50^***^	*z* = 4.5^***^
**PSEUDOWORDS**					
Accuracy/20	17.5 (1.9)	0.310	11.1 (3.9)	−2.40^*^	*t*_(30)_ = −5.6^***^
Speed (s)	24.3 (6.0)	0.08	57.3 (25.7)	−3.10^**^	*z* = 4.8^***^

a *Individual z-scores (one-tailed) computed according to age-matched norms (Bosse and Valdois, 2009)*.

b *For speed measures, non-parametric Mann-Whitney U-tests were used (z statistics reported)*.

* *p < 0.05*,

** *p < 0.01*,

*** *p < 0.001*.

### Phonological skills and VA span skills in the dyslexic group

Table 2 presents the performance of the dyslexic group regarding their phonological and VA Span skills. The dyslexic group was significantly worse at repeating pseudowords compared to the age-matched norm, which illustrated poor phonological short-term memory skills (*z* = −1.84, *p* < 0.05). In the CCV phonemic deletion task, the dyslexic group tended to exhibit poorer performance compared to the norm (*z* = −1.54, *p* = 0.06). On the visual control task of single letter identification, no deficit was found at any of the presentation times (33 ms: *z* = −0.49, 50 ms: *z* = −0.59, 67 ms: *z* = −0.56, 84 ms: *z* = −0.67 and 110 ms: *z* = −0.67, all *z*s n.s.), neither on the overall performance (104.4 letters identified out of 150 (±34), *z* = −0.56, n.s.). On the whole report task, the dyslexic group accurately reported 65.6% (±16.3) of the letters on average, indicating a deficit on that task (*z* = −1.81, *p* < 0.05). On the partial report task, as a group, the dyslexic children did not exhibit any deficit, reporting accurately 78% (±16.3) of the cued letters (*z* = −0.88, n.s.).

**Table 2:** **Characteristics of the dyslexic group (*n* = 16)**.

	**M (*SD*)**	**Range**	***Z* score**
**PHONOLOGY**			
Pseudoword repetition (%)^a^	45.5 (20)	19–79	−1.84^*^
CCV deletion (%)^a^	51.0 (27)	0–100	−1.54, *p* = 0.06
**VISUAL ATTENTION SPAN^b^**			
Whole report task (%)	65.6 (16.3)	35–91	−1.81^*^
Partial report task (%)	78.0 (13)	50–92	−0.88 n.s.

a *z scores computed from the age-matched norms of Sprenger-Charolles et al. (2005)*.

b *z scores computed from the age-matched norms of Bosse and Valdois (2009)*.

* *p < 0.05*.

### Search tasks

#### Search performance differences between the control and dyslexic groups

**Figure 2:**
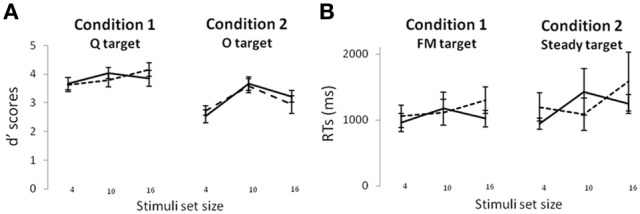
**Visual d′ scores (A) and auditory RT scores (B) in the control (solid line) and dyslexic (dotted line) children.** Standard error bars are depicted.

First, in the visual modality (Figure 2A), no effect involving the group was found on d' scores (all *Fs* < 1), indicating that there was no group difference on visual target detection sensitivity across all experimental conditions. In the auditory modality (Figure 2B), no effect was found on RTs (all *Fs* < 1) indicating that none of the factors (including the group) influenced the time to press the response button after the auditory configuration presentation.

**Figure 3:**
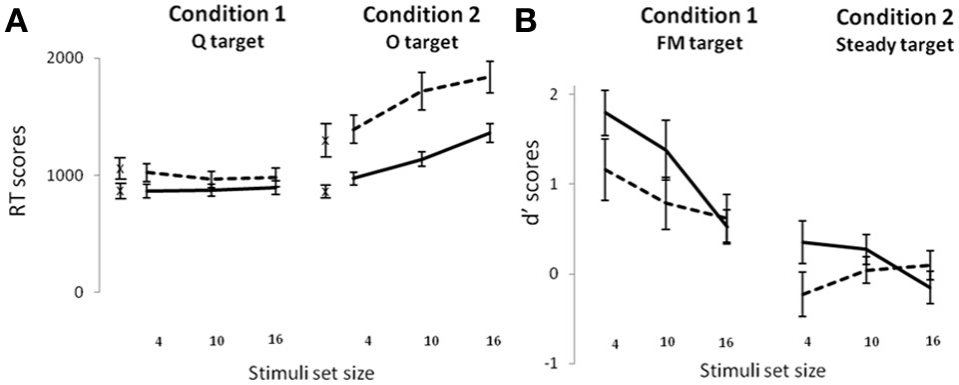
**Visual RT scores (A) and auditory d′ scores (B) in the control (solid line) and dyslexic (dotted line) children.** Crosses represent the intercept values for the visual task. Standard error bars are depicted.

In the visual task (Figure 3A), there was a main effect of group on RTs [*F*_(1, 30)_ = 4.90, *p* = 0.034] that was modulated by the condition [*F*_(1, 30)_ = 5.6, *p* = 0.02], and that showed that dyslexic children were slower than control children in the O target condition (*post-hoc*: *p* = 0.03 but) but not in the Q target condition (*post-hoc*: *p* > 0.9). There was also a main effect of condition [*F*_(1, 30)_ = 119.05, *p* < 0.001] and stimulus set size [*F*_(2, 60)_ = 22.3, *p* < 0.001). These two factors interacted with each other [*F*_(2, 60)_ = 26.2, *p* < 0.001] indicating that the smaller the number of distracters, the faster the response, but only for the O target condition (*post-hoc*: all *ps* < 0.001; Q target condition, all *ps* > 0.1). RTs differences across stimulus set sizes in the O target condition could be explained by changes in participant's criteria (speed-accuracy tradeoff): Average d' scores and RTs indeed positively correlated in the whole sample (*r* = 0.34, *p* < 0.05) suggesting that the worse target sensitivity the child showed, the faster at responding they were. Importantly, since the two groups showed similar d' scores for all visual experimental conditions (see Figure 2A), speed-accuracy tradeoff variations between groups could not explain the aforementioned differences on RTs. As a follow-up of these significant effects on RTs, we computed intercept and slope values of the search functions. The group difference found in the O target condition on RTs was accompanied by a group difference on the search function intercepts (controls: 860 ± 213 ms; dyslexics: 1260 ± 520 ms; *t*_(30)_ = −2.7, *p* < 0.05), but not on the slopes (controls: 32 ± 18 ms/item; dyslexics: 37 ± 32 ms/item; *t* < 1). In the Q target condition, control and dyslexic children presented identical slopes (respectively, 2 ± 8 ms/item and −3 ± 16 ms/item, *t*_(30)_ = 1.2, *p* > 0.05) and intercepts (respectively, 860 ± 245 and 1037 ± 322 ms, *t*_(30)_ = −1.7, *p* > 0.05).

In the auditory task (Figure 3B), no main effect of group was found [*F*_(1, 30)_ = 2.14, *p* = 0.15] on d′ measures. There was a main effect of condition [*F*_(1, 30)_ = 35, *p* < 0.001] illustrating that participants were better in the FM target condition than in the Steady target condition. There was also an effect of stimulus set size [*F*_(2, 60)_ = 5.3, *p* < 0.01] showing that target detection performance was better for stimulus set size of four than 16 (*post-hoc* test: *p* < 0.01, other *ps* > 0.05). A condition by stimulus set size interaction [*F*_(2, 60)_ = 4.2, *p* = 0.02] revealed that the difference between the set sizes of 4 and 16 was true for the FM target condition (*post-hoc* test: *p* < 0.001), whereas in the Steady target condition, detection was equally hard for all set sizes (*post hoc* tests: all *ps* > 0.10). The condition by set size interaction was similar between groups (*F* < 1). Lastly, there was an interaction between group and stimulus set size [*F*_(2, 60)_ = 3.4, *p* = 0.04] showing that control children benefited of being presented with four compared to 16 stimuli (*post-hoc* test: *p* < 0.005) whereas dyslexic children did not, and this was not modulated by the condition (*F* < 1).

#### Search performance differences between dyslexic subgroups

In order to examine to what extent individual significant VA Span disorders in the dyslexic group were linked to search deficits, we ran subsequent subgroup analyses. We selected all the dyslexic children with a VA Span deficit, i.e., impaired on both the whole and partial report tasks (VASpan subgroup; all individual *z*-scores < −1.65, *n* = 6). We further selected all the dyslexic children with no impairment on any of the two report tasks (noVASpan subgroup: individual *z*-scores > −0.9, *n* = 6). Note that the four remaining dyslexic children exhibited poor performance on only one of the two report tasks and were not included in any of the subgroups. Both of the two subgroups presented the same reading delay compared to the level expected for their age (42 months for the VASpan subgroup and 48 months for noVASpan subgroup), which indicated similar impaired reading level in the two dyslexic subgroups (Mann Whitney *U*-test: *z* = 1.26 *p* > 0.05). Both dyslexic subgroups were impaired on phonological short term memory (VASpan: *z* = −2.1, *p* = 0.018; noVASpan: *z* = −1.9, *p* = 0.029), but only the noVASpan subgroup was significantly impaired on phonemic awareness (noVASpan: *z* = −1.7, *p* = 0.045; VASpan: −1.2, *p* = 0.11 n.s.). Kruskal-Walis tests were conducted with group as a between subject factor (VASpan, *n* = 6; noVASpan, *n* = 6; controls, *n* = 16) on the average performance in the visual O target condition and in the auditory FM target condition. On auditory search, the three groups presented similar performance overall [*H*_(2)_ = 0.8 *p* = 0.66]. On visual search, there was a main effect of group on RTs [*H*_(2)_ = 11.1 *p* < 0.005] that indicated slower target detection for the VASpan subgroup compared to the control group (multiple comparisons on mean ranks: *p* < 0.005, all other *ps* > 0.05). Note that no group effect was found on visual d' scores on the O target condition [*H*_(2)_ = 0.59 *p* = 0.77], suggesting no speed-accuracy tradeoff differences across groups. Although no group effect was found on the slope values [*H*_(2)_ = 2.1 *p* = 0.33], a group effect was found on the intercept values [*H*_(2)_ = 6.8 *p* = 0.034], which revealed higher intercepts for the VASpan group than the control group (multiple comparisons on mean ranks: *p* = 0.04, all other *ps* > 0.05).

### Partial correlations analyses

#### Within the whole sample

Following the results reported in section Search Tasks, an average d' obtained on the O target condition was controlled for in the correlations involving RTs for visual search in order to neutralize speed-accuracy tradeoff between participants. None of the reading measures correlated with any of the different search measures neither in the Q target condition for the visual task, nor in the Steady target condition for the auditory task. Therefore, all the subsequent analyses will focus on performance on the O target condition and the FM target condition.

Performance on these critical conditions correlated with each other (−0.33, *p* < 0.05, one-tailed, based on the *a priori* hypothesis of an amodal pool of resources for simultaneous processing, cf. Lallier et al., 2012) suggesting that they tapped into the same pool of amodal resources. As shown in Table 3, all reading *z*-scores significantly correlated with visual RTs, illustrating that the better reading (accurate and fast) the faster the search, for all stimulus set sizes. The reading scores also correlated with the intercept measures, but not with the slope measures, suggesting that the greater the intercept the poorer the reading skills. In the auditory modality, search performance (in particular for a set size of 10) significantly correlated with reading speed for all types of items and with real words only (regular and irregular) regarding accuracy, i.e., the higher the d', the faster and better the reading.

**Table 3:** **Correlation coefficients between reading *z*-scores and search tasks in the whole sample (*n* = 32)**.

	**REG_Acc**	**REG_T**	**IRR_Acc**	**IRR_T**	**PW_Acc**	**PW_T**
**VISUAL SEARCH O TARGET**						
RT—SSS(4)	−0.43^**^	−0.51^***^	−0.57^***^	−0.48^***^	−0.63^***^	−0.32^*^
RT—SSS(10)	−0.49^***^	−0.57^***^	−0.59^***^	−0.54^***^	−0.64^***^	−0.43^***^
RT—SSS(16)	−0.39^*^	−0.41^*^	−0.53^***^	−0.38^*^	−0.47^**^	−0.39^*^
RT—AVG	−0.46^***^	−0.53^***^	−0.60^***^	−0.50^***^	−0.61^***^	−0.40^*^
Slope	−0.01	0.07	−0.04	0.08	0.14	−0.15
Intercept	−0.40^*^	−0.49^***^	−0.52^***^	−0.47^***^	−0.62^***^	−0.27
**AUDITORY SEARCH (d′) FM TARGET**						
SSS(4)	0.37^*^	0.30^*^	0.31^*^	0.17	0.10	0.36^*^
SSS(10)	0.34^*^	0.41^*^	0.33^*^	0.35^*^	0.27	0.36^*^
SSS(16)	0.29	0.08	0.02	0.04	0.18	0.09
AVG	0.41^*^	0.35^*^	0.30	0.25	0.23	0.35^*^

*REG, regular words; IRR, irregular words; PW, pseudowords; Acc, accuracy; T, time; SSS, stimulus set size; AVG, average measure of the search performance across the three stimuli set sizes*.

* *p < 0.05*,

** *p < 0.01*,

*** *p < 0.005, one-tailed based on the a priori hypothesis of a relation between reading deficits and poor search performance*.

#### Within the dyslexic sample

We further ran partial correlation analyses in the dyslexic group in order to determine whether a low search performance in both modalities would contribute to their reading and/or cognitive deficits (i.e., phonological or VA Span difficulties). As seen in Table 4, auditory (d') and visual (RTs) search performance correlated with each other indicating that the higher the RTs in the visual task, the lower the d' score in the auditory task. Moreover, the higher the visual intercepts, the poorer the auditory search performance for a stimulus set size of 10.

**Table 4:** **Partial correlation coefficients visual and auditory search performance in the dyslexic sample (*n* = 16)**.

	**Auditory search (d′) FM target**			
	SSS(4)	SSS(10)	SSS(16)	AVG
**VISUAL SEARCH O TARGET**				
RT—SSS(4)	−0.07	−0.68^***^	−0.30	−0.35
RT—SSS(10)	−0.05	−0.79^***^	−0.52^*^	−0.52^*^
RT—SSS(16)	−0.22	−0.75^***^	−0.68^***^	−0.65^**^
RT—AVG	−0.06	−0.79^***^	−0.52^*^	−0.53^*^
Slope	−0.37	0.01	−0.43	−0.33
Intercept	0.14	−0.60^*^	−0.17	−0.24

*SSS, stimulus set size; AVG, average measure of the search performance across the three stimuli set sizes*.

* *p < 0.05*,

** *p < 0.01*,

*** *p < 0.005, one-tailed*.

**Figure 4:**
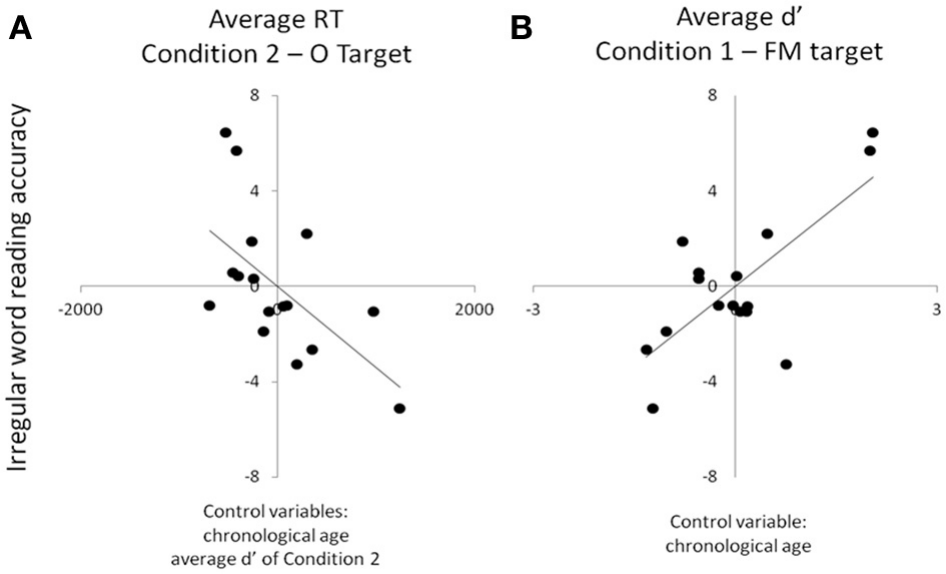
**Scatterplots depicting the partial correlations between irregular word reading accuracy (*y* axes) and visual (A) and auditory (B) search performance among the dyslexic children.** For each panel, individual residual scores are represented, which stem from the two correlations between the factor(s) controlled for and (i) search performance, as well as (ii) irregular word reading accuracy.

Poor search skills of dyslexic children were associated with their poor reading skills. Visual search RTs and intercepts correlated with pseudoword and irregular word reading accuracy and auditory search d′ scores correlated with real word reading accuracy (regular and irregular) and reading speed for all items (Table 5). In particular, both visual (i.e., set sizes of 4, 10, and 16, average measure, intercepts) and auditory search performance (set size of 10 in particular) correlated strongly with irregular word accuracy (cf. Table 5; Figure 4). Visual slope values did not correlate with any of the reading measures.

**Table 5:** **Partial correlation coefficients between reading scores and search performance in the dyslexic sample (*n* = 16)**.

	**REG_Acc**	**REG_T**	**IRR_Acc**	**IRR_T**	**PW_Acc**	**PW_T**
**VISUAL SEARCH O TARGET**						
RT— SSS(4)	−0.31	0.24	−0.69^***^	0.32	−0.56^*^	0.19
RT—SSS(10)	−0.34	0.34	−0.79^***^	0.41	−0.48^***^	0.31
RT—SSS(16)	−0.18	0.11	−0.66^**^	0.15	−0.28	0.09
RT—AVG	−0.30	0.26	−0.76^***^	0.33	−0.48^*^	0.23
Slope	0.21	−0.19	0.14	−0.25	0.42	−0.15
Intercept	−0.32	0.25	−0.64^**^	0.33	−0.58^*^	0.20
**AUDITORY SEARCH (d′) FM TARGET**						
SSS(4)	0.46^*^	−0.21	0.46^*^	−0.07	−0.02	−0.26
SSS(10)	0.53^*^	−0.51^*^	0.84^***^	−0.48^*^	0.32	−0.48^*^
SSS(16)	0.40	−0.10	0.52^*^	−0.10	0.24	0.09
AVG	0.57^*^	−0.33	0.74^***^	−0.26	0.20	−0.34

*REG, regular words; IRR, irregular words; PW, pseudowords; Acc, accuracy; T, time; SSS, stimulus set size; AVG, average measure of the search performance across the three stimuli set sizes*.

* *p < 0.05*,

** *p < 0.01*,

*** *p < 0.005, one-tailed based on the a priori hypothesis of a relation between reading deficits and poor search performance*.

**Figure 5:**
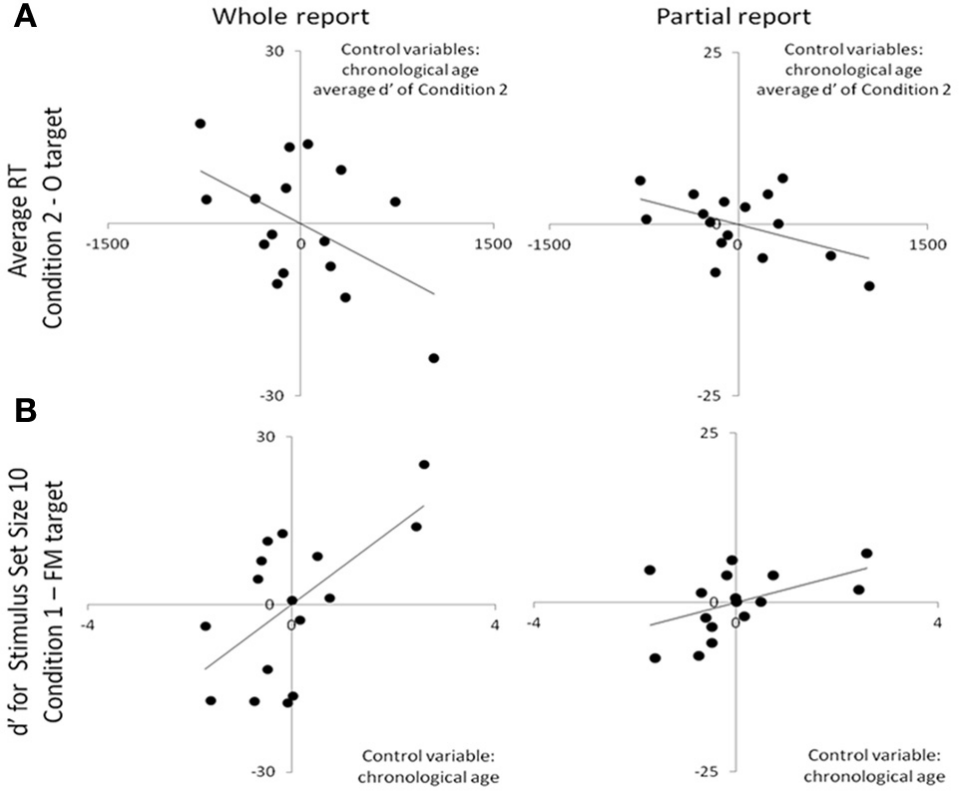
**Scatterplots depicting the partial correlations between VA Span skills (whole and partial report, *y* axes) and visual (A) and auditory (B) search performance (*x* axes) among the dyslexic children.** For each panel, individual residual scores are represented, which stem from the correlations between the factor(s) controlled for and (i) VA Span skills, as well as (ii) search performance.

Lastly, reduced VA Span skills were found to be associated with both poor visual (RTs and intercepts) and auditory (set size of 10) search performance (Table 6; Figure 5). No relation was found between search performance and any of the two phonological scores.

**Table 6:** **Partial correlation coefficients between cognitive skills and search performance in the dyslexic sample (*n* = 16)**.

	**VA Span**		**Phonology**	
	**PARTIAL**	**WHOLE**	**PSTM**	**PA**
**VISUAL SEARCH O TARGET**				
RT—SSS(4)	−0.37	−0.48^*^	−0.13	0.38
RT—SSS(10)	−0.47^*^	−0.60^*^	−0.12	0.34
RT—SSS(16)	−0.52^*^	−0.31	−0.05	0.26
RT—AVG	−0.48^*^	−0.51^*^	−0.11	0.35
Slope	−0.14	0.26	0.12	−0.20
Intercept	−0.30	−0.47^*^	−0.14	0.38
**AUDITORY SEARCH (d′) FM TARGET**				
SSS(4)	0.04	0.28	0.33	0.11
SSS(10)	0.48^*^	0.61^*^	0.30	−0.20
SSS(16)	0.32	0.15	0.13	0.05
AVG	0.32	0.43	0.32	0.01

*PSTM, phonological short term memory; PA, phonological awareness; SSS, stimulus set size; AVG, average measure of the search performance across the three stimuli set sizes*.

* *p <0.05*.

## Discussion

In the present study, we showed that dyslexic individuals are impaired in visual “serial” search paradigms. Dyslexic children were indeed slower than the control group to detect the letter O among Qs. This deficit was accompanied by a search function characterized by prolonged intercepts for the dyslexic group than the control group in the absence of difference on the search slope. Moreover, auditory search abilities were measured for the first time in children with and without developmental dyslexia. The condition where children had to detect a steady sound among FM sound distracters led to very low performance, suggesting that this task was too difficult for both the controls and the dyslexic groups. In both conditions, the control children's performance was enhanced when few auditory distracters were present, suggesting that stimulus set size influenced their perceptual attentional auditory load. No such modulation was observed in the dyslexic children, which might indicate that their auditory perceptual attention load is already “*at threshold*” for the processing of few stimuli, and might reflect a limitation of attentional resources allocated to auditory simultaneous processing. We will return to this point later. In favor of the amodality of perceptual attention at play in search mechanisms, fast visual “serial” search (O target) correlated significantly with good sensitivity to detect FM targets both in the whole group and within the dyslexic group of children.

Slow visual “serial” search and high intercepts were significantly associated with poor VA Span skills in the dyslexic group. More specific analyses of the individual cognitive profiles of the dyslexic children showed that only the children with a significant VA Span deficit (i.e., impaired on both the whole and the partial report tasks) exhibited slower RTs and higher intercepts on the visual “serial” search compared to controls. This result strongly suggests that the factor at play in both reading and our visual search task is linked to simultaneous visual attention, i.e., the number of visual elements that can be processed in parallel in one fixation. Importantly, we found no correlation between visual search skills and either phonological short term memory or phonemic awareness skills of the dyslexic children. Moreover, the dyslexic group with no VA Span impairment showed no deficit on the visual search task despite of being the only group impaired on phonemic awareness. This dissociation between phonological skills and visual search performance in the dyslexic group suggests that deficits in the “serial” search condition (O target) is unlikely to be driven by serial processing difficulties such as sluggish visual attentional shifting skills previously found to relate to poor phonological skills (Lallier and Valdois, 2012 for a review). This idea finds additional support in both the absence of group effect on the slope values and the absence of correlation between reading, VA Span and these slope values. Indeed, search slope values are thought to relate to serial attentional components of the search (Wolfe and Horowitz, 2008). In our study, the dyslexic children—in particular the subgroup with simultaneous visual processing deficits (VASpan subgroup)—did not exhibit any deficit in the visual sequential processing skills required for an efficient search (i.e., absence of atypically high slope values). This is reminiscent of the results of Lallier et al. (2010a,b,c) which show that simultaneous and sequential visual attentional deficits dissociate in developmental dyslexia. The dyslexic children in our study were rather impaired on components such as those determining the intercept of the search function. Intercept values have been related to factors linked to processes preceding or following search mechanisms *per se* (Woodman et al., 2001). The intercept values of the dyslexic children might therefore have been constrained by visual mechanisms that influenced their response, and that were possibly at play before the start of the attentional serial search (Jolicoeur and Dell'Acqua, 1999; Wolfe and Horowitz, 2008). We will discuss later on how such visual mechanisms could relate to VA Span skills.

In further support for the significant contribution of VA Span disorder to the visual search deficits of dyslexic children, we reported that their visual “serial” search performance (RTs and intercepts) and reading accuracy significantly correlated and especially strongly so for irregular words. In opaque languages like French, it is impossible to correctly read an irregular word using the most frequent grapheme-to-phoneme conversion rules: the only way to be accurate is to retrieve the phonological lexical form automatically from the whole-word visual form whose encoding depends on VA Span skills (Ans et al., 1998; Bosse and Valdois, 2009). Our results in the auditory modality were less clear due to an absence of difference between both the dyslexic and control groups as well as the VASpan and NoVASpan dyslexic subgroups. Still, like in the visual modality, a strong correlation was found between irregular word reading accuracy and auditory search performance (stimulus set size of 10) within the dyslexic group, suggesting that auditory simultaneous attention may also be important for lexical processing in dyslexia. For the irregular word list, dyslexic children had to read each item aloud, hence retrieve its phonological lexical form. Would auditory simultaneous attention mediate the access of auditory whole-word knowledge?

Studies have shown some links between auditory whole-word knowledge and the degree of divided attention engaged in a task. For example, dual-tasking seems to enhance the reliance of lexical knowledge strategies used in speech perception tasks: Mattys and Wiget (2011) showed that the reliance on lexical knowledge was stronger in high cognitive load settings simulating adverse speech perception conditions (dual task, divided attention) than in low cognitive load settings (see also Mattys et al., 2009)^3^. In the present study, the correlation between visual and auditory search performance and irregular word reading accuracy in the dyslexic group indicates that children with better phonological and orthographical whole-word knowledge (and good access to them) were those who could monitor better random increases of the load of perceptual attention for auditory search. According to (Mattys and Wiget, 2011, Experiment 6), the strong lexical knowledge reliance for speech processing in high cognitive load settings might stem from the need to cope with the sensory degradation of *temporal cues* important for phoneme identification. Similarly, Casini et al. (2009) showed that vowel duration was underestimated when participants had to perform a dual task. According to the hypothesis that the estimation of speech units' duration relies on registering the number of “temporal pulses” accumulated during speech unit intervals (e.g., Coull et al., 2004), Casini et al. (2009) proposed that sharing attentional resources between simultaneous tasks (or simultaneous stimuli in the present study) decreases the sampling rate allocated to each task (or stimulus). Such phonemic sampling reduction would thus lead to miss some pulses within phonemic intervals and the underestimation of their temporal features.

In favor of Casini et al. (2009)'s hypothesis and the hypothesis of a permanent “*dual-task-like*” mode of dyslexic individuals, Vandermosten et al. (2010, 2011) showed that dyslexic children exhibit poor phonemic identification skills relying on temporal cues. Lehongre et al. (2011) also showed that dyslexic adults exhibited less oscillatory neural entrainment than skilled readers at the phonemic sampling rate (30 Hz) in the left hemisphere, which further correlated to slow rapid automatized naming skills (phonological whole-word forms retrieval). This last result supports our idea that auditory whole-word knowledge relies on the quality of both phonemic-rate sampling (cf. Poeppel, 2003) and the monitoring of random increases of auditory perceptual attentional load (cf. Figure 5). The correlations highlighted between auditory/visual search and VA span skills suggest that dyslexic children with low VA Span skills may suffer from a permanent high perceptual load hindering visual and auditory processing. This should be particularly true when *several* stimuli have to be attended and encoded (like in “serial” search) since we did not report different intercepts between the dyslexic and control children when the attentional focus is automatically directed to *the* relevant target stimulus (such as in “parallel” search). In support of this idea, Woodman et al. (2001) reported an increase of the intercept but not the slope values of the visual “serial” search functions (like the performance of our dyslexic group) when participants had to maintain several visual elements in memory whilst performing the search. A VA Span reduction might therefore have a negative impact on perceptual attentional processing similar to the one generated by a cognitive overload stemming from dual-tasking.

Interestingly, the auditory perceptual load generated by the simultaneous presentation of 10 stimuli was found to correlate with VA Span skills, intercept values and reading skills. Why this particular “signal-to-noise ratio” may be relevant for literacy development is an open question, but some studies looking at speech-in-noise deficits in developmental dyslexia could shed light on it (Ziegler et al., 2009; Dole et al., 2012). In particular, we found that auditory search performance was unrelated to phonological awareness (see also Lallier et al., 2012) which is supported by studies that show a relative independence between speech-in-noise deficits and other phonological deficits (Robertson et al., 2009; Ziegler et al., 2009; Messaoud-Galusi et al., 2011; Berent et al., 2012; Dickie et al., 2012). Speech-in-noise skills of dyslexic children also seem to dissociate from slow rate dynamic auditory processing linked to phonological awareness (Poelmans et al., 2011). Weak auditory entrainment to slow auditory frequencies (delta and theta) within speech streams—and critical for rhythm extraction—has been proposed as a cause of phonological awareness deficits in dyslexia (Goswami, 2011; Goswami et al., 2013; Hämäläinen et al., 2012). This weak auditory oscillatory entrainment at slow frequency bands might thus explain sluggish auditory attentional shifting, which appears to be restricted to dyslexia associated with phonological disorders (see Lallier et al., 2013), but is less likely to explain poor simultaneous processing abilities in auditory search. It is noteworthy that in the present study, we did not observe any significant deficit of the dyslexic group on our auditory search task. Although no strong conclusion about the role of auditory search in developmental dyslexia can be drawn at this point, the significant relationships highlighted between auditory search performance and reading skills as well as VA Span skills can still shed light on what this role might be.

Overall, we point out that different auditory perceptual attentional factors might contribute independently^4^ to stable sound representations build-up (Hornickel and Kraus, 2013) and reading development. One of them could relate to speech processing in particular in high perceptual load situations (i.e., speech-in-noise, auditory search) and lexical knowledge, and would be linked to the simultaneous dimension of auditory processing (high frequency sampling). Another one would tap into slow modulations which are important for phonological awareness acquisition and that are carried by speech rhythm: this slow frequency sampling would tap into the sequential dimension of auditory processing^5^. We propose that a similar model could *a priori* hold true for the visual modality since perceptual attention deficits on one processing dimension (sequential or simultaneous) generally co-occur in audition and vision in the same dyslexic participants (Lallier et al., 2009, 2010a,c, 2012). We suggest that visual perceptual attention critical for reading acquisition requires both sequential (slow) and simultaneous (fast serial) mechanisms. First, a sequential visual attention mechanism would guide the attentional focus to engage and disengage over orthographic sequences, explaining sluggish visual attentional shifting and phonological disorders in dyslexia (Hari and Renvall, 2001; Facoetti et al., 2008; Lallier et al., 2009, 2010a,b). This visual mechanism would possibly trigger saccades in reading (Belopolsky and Theeuwes, 2009) and tap into parvocellular, hence relatively slow, temporal processing (Vidyasagar, 2004). A second mechanism, engaged in visual “serial” search (O target) would be in charge of screening the orthographic chunks falling under fixation and could be monitored by the magnocellular pathway, hence, characterized by a very high rate serial processing (Vidyasagar, 2004). The present results suggest that this second mechanism might be in part modulated by VA Span skills. Future studies will explore whether and how VA Span skills are monitored by a high frequency oscillatory visual system (gamma band) and whether this system dissociates from or depends on its coupling with a slow frequency oscillatory visual system (delta/theta bands).

## Conclusion

In the present study, we assessed the search performance of a group of dyslexic children across the visual and the auditory modalities. Dyslexic children were slower than control children on the visual “serial” search condition only, which was accompanied by search function intercepts (but not slopes) that were higher in the dyslexic group than the control group. Despite the absence of deficit of the dyslexic group on the auditory search task, we showed that poor VA Span skills correlated to poor search performance not only in the visual but also in the auditory modalities. These results suggest that dyslexic children with a VA Span disorder may be under a permanent high perceptual load that hinders visual and auditory processing in particular in situations where several elements must be encoded simultaneously. Our results also suggest that limitations in simultaneous perceptual attention may preferentially affect the development of lexical reading (e.g., irregular word reading) and possibly the build-up of some phonological processes (first at the phoneme level, with consequences for auditory whole-word forms access). Finally, we stress the importance of taking into account the heterogeneity of the reading disorders at the cognitive level (e.g., phonological awareness, VA Span), since various time scales of processing might have different and potentially independent roles in literacy acquisition, and lead to different subtypes of developmental dyslexia.

### Conflict of interest statement

The authors declare that the research was conducted in the absence of any commercial or financial relationships that could be construed as a potential conflict of interest.

## Footnotes

^1^The theoretical nuance between the two theories is that Vidyasagar proposes that these visual attentional resources are serial (since it would be governed by the dorsal stream) whereas the VA Span hypothesis assumes that they are parallel (since they would be subtended by brain areas playing a role attention devoted to multi-element processing: Peyrin et al., 2012). Another difference between the two theories is that Vidyasagar defines the visual attentional resources in terms of spatiality whereas in the VA Span hypothesis, they are defined in terms of quantity.

^2^In Cusack and Carlyon (2003, Experiment 3), the temporal window was 2 s long. We shortened this temporal window on purpose to favor the simultaneous processing of the tones.

^3^Even if we did not manipulate the cognitive load in our search tasks, perceptual load was. Perceptual and cognitive loads may further not be that independent in dyslexia since Lallier et al. (2012) showed that dyslexic children's scores on a dichotic listening dual-task (cognitive load) correlated with VA Span skills (perceptual attention load).

^4^Since the sampling frequencies characterizing speech streams are coupled and modulate each other, the independence of their respective contribution to reading and dyslexia may be relative and remains to be quantified.

^5^Most of the speech-in-noise deficits assessed in dyslexia reflect problems at lexical, syllabic or phonemic levels. Future studies should examine whether “speech streams”-in-noise perception shares any mechanisms with rhythm perception, phonological awareness and slow rate auditory entrainment.
